# METNET: a phase II trial of metformin in patients with well-differentiated neuroendocrine tumours

**DOI:** 10.3332/ecancer.2022.1421

**Published:** 2022-07-04

**Authors:** João Glasberg, Aley Talans, Thomás Rivelli Giollo, Débora Zachello Recchimuzzi, João Evangelista Bezerra Neto, Rossana Veronica Mendonza Lopez, Paulo Marcelo Gehm Hoff, Rachel P Riechelmann

**Affiliations:** 1 Department of Clinical Oncology, Instituto do Câncer do Estado de São Paulo, Faculdade de Medicina da Universidade de São Paulo – FMUSP, São Paulo, Brazil; 2 Post Graduate Program, Faculdade de Medicina da Universidade de São Paulo, Brazil; 3 Department of Medical Radiology, Fleury Group, São Paulo, Brazil; 4 Department of Medical Radiology, Hospital Israelita Albert Einstein, São Paulo, Brazil; 5 Center for Translational Research in Oncology, Instituto do Câncer do Estado de São Paulo, Faculdade de Medicina da Universidade de São Paulo – FMUSP, São Paulo, Brazil; 6 Department of Clinical Oncology, AC Camargo Cancer Center, São Paulo, Brazil

**Keywords:** neuroendocrine tumours, metformin, cancer treatment

Thomás Rivelli Giollo is corrected to Thomás Giollo Rivelli

